# Sudden Diplopia Two Months After a Blowout Fracture: A Case Report of Orbital Tissue Adhesion Associated With Acute Sinusitis

**DOI:** 10.7759/cureus.77659

**Published:** 2025-01-19

**Authors:** Shohei Tsuji, Yu Hosokawa, Hiroki Sato, Kazuyuki Tokioka, Tetsuo Ikezono

**Affiliations:** 1 Otorhinolaryngology, Saitama Medical University, Saitama, JPN; 2 Plastic and Reconstructive Surgery, Saitama Medical University, Saitama, JPN

**Keywords:** diplopia, enophthalmos, orbital floor blowout fracture, persistent diplopia, scar adhesion

## Abstract

Blowout fractures (BOFs) frequently result in early-onset diplopia, which often resolves spontaneously. Delayed diplopia is rare, and there are no reported cases of its sudden onset following a prolonged asymptomatic period. Persistent diplopia significantly impacts quality of life. BOF-induced late-onset diplopia is rare. To date, no cases of late-onset or acute-onset diplopia have been reported, underscoring its exceptional rarity. This report discusses a unique case of sudden diplopia two months post BOF, which improved after surgical treatment.

A 57-year-old man presented with a history of BOF without initial diplopia. Two months post injury, he developed acute diplopia on an upward gaze. Computed tomography revealed an extensive orbital floor fracture with herniation into the maxillary sinus and odontogenic maxillary sinusitis. The late-onset diplopia was believed to be caused by adhesions of the tissues surrounding the inferior rectus muscle due to the spread of maxillary sinus inflammation into the orbit. Surgery was performed 154 days after the trauma, which included endoscopic sinus surgery and transconjunctival orbital floor repair. Intraoperatively, the maxillary sinus mucosa was severely edematous and prone to bleeding. Adhesions between the tissue around the inferior rectus muscle and orbital floor were identified and sharply dissected. Surgical intervention effectively resolved enophthalmos and significantly improved diplopia.

This case of BOF is extremely rare, as diplopia developed both late and suddenly. Although 154 days had passed since the trauma, the patient showed significant improvement following surgical treatment. The cause of the late and acute-onset diplopia is believed to be the spread of inflammation from odontogenic maxillary sinusitis into the orbit, resulting in adhesions in the tissues surrounding the inferior rectus muscle. Delayed onset of diplopia in BOF may be an early symptom of intraorbital inflammation. In cases of extensive BOF accompanied by paranasal sinusitis with severe inflammation, we believe that several months of follow-up observation is necessary to prevent late complications.

## Introduction

The causes of blowout fractures (BOFs) are believed to involve either a sudden increase in intraorbital pressure (the hydraulic mechanism) [1] or the transmission of impact through bone conduction from the orbital rim to the orbital floor (the buckling mechanism) [2].

This is a common facial injury frequently encountered by otolaryngologists and plastic surgeons. The primary complications include diplopia, enophthalmos, and sensory paralysis [3]. BOF frequently results in early-onset diplopia, which often resolves spontaneously. If diplopia does not improve two weeks after the trauma, surgery will be indicated [4], but if there is no diplopia, the patient will be observed. Therefore, such cases often end up being followed up. Since the persistence of diplopia significantly affects patients' quality of life (QOL), numerous studies have been conducted on the topic [5].

Delayed diplopia is rare, and there are no reported cases of its sudden onset following a prolonged asymptomatic period. This report discusses a unique case of sudden diplopia two months post BOF, which improved after surgical treatment.

## Case presentation

A 57-year-old man with no history of nasal surgery visited an ophthalmology clinic after falling into a bamboo fence and experiencing swelling around his eyelids. Initial findings included a lower eyelid laceration without diplopia or ocular motility impairment. There was no diplopia observed during the previous doctor's examination 40 days after the trauma.

**Figure 1:**
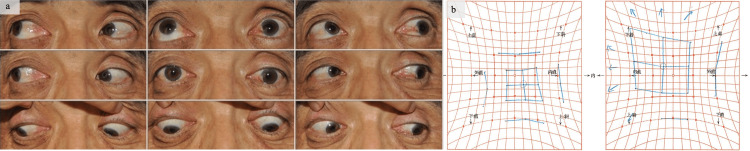
Eye movement and enophthalmos findings at the initial examination at our hospital. (a) The left eye appears noticeably sunken in the frontal view. There is a movement restriction of the left eye when looking upward. (b) Results of the Hess test: the patient exhibits impaired upward movement of the left eye. We obtained written consent from the patient to publish the photo in an open-access journal.

Two months after the trauma, the patient suddenly noticed diplopia while looking at a traffic light and visited a general hospital. He later came to our hospital for further examination and treatment. Examination revealed left enophthalmos and restricted upward gaze. Hess screen test confirmed significant restrictions in ocular motility (Figure 1).

**Figure 2:**
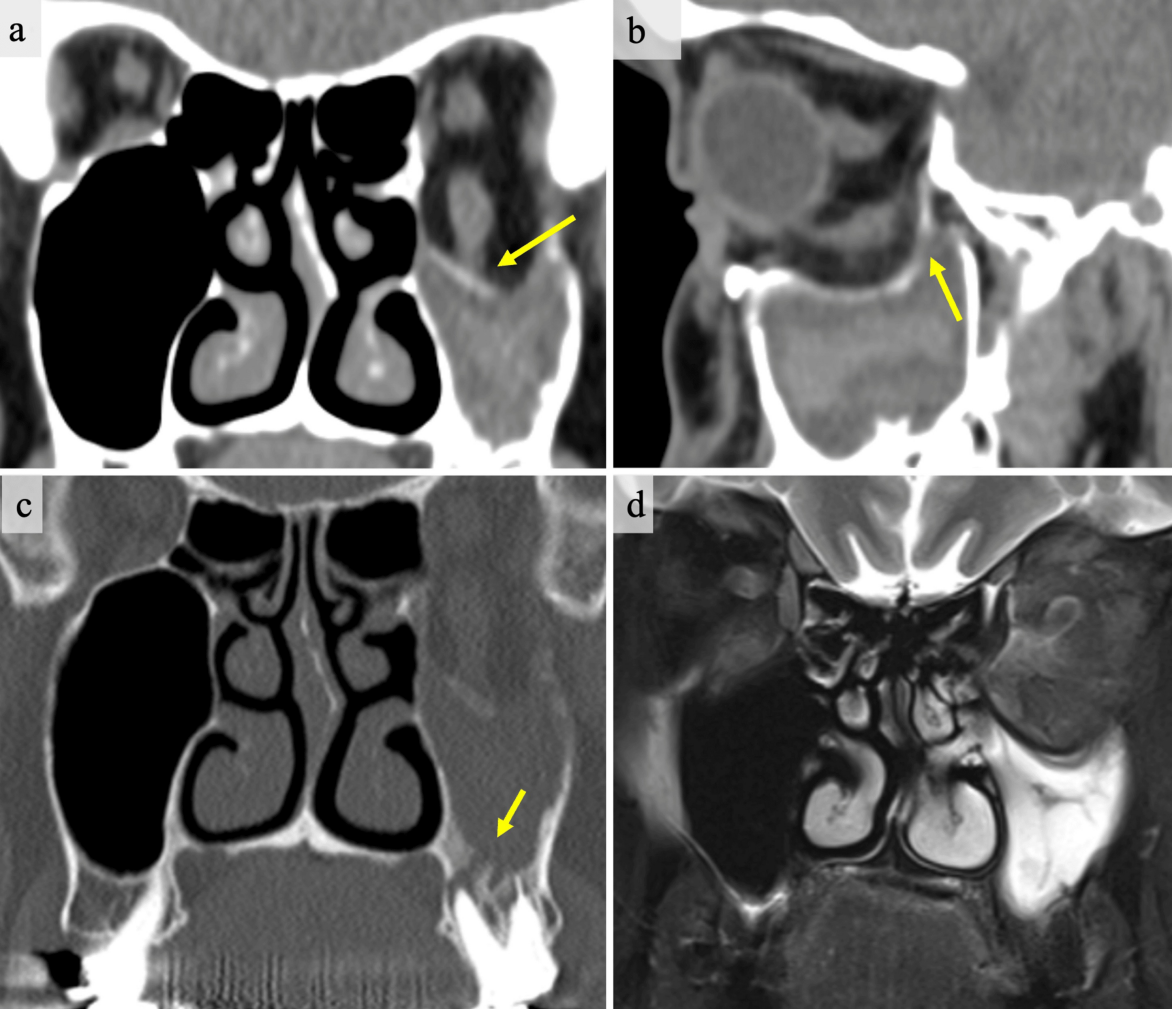
CT and MRI findings at the initial examination in our hospital (two months after the trauma). (a) A coronal CT scan shows significant displacement of the orbital contents, including the inferior rectus muscle. Adhesion of the tissue around the inferior rectus muscle is suspected at the point indicated by the arrow. (b) A sagittal CT scan reveals an extensive orbital floor fracture extending both anteriorly and posteriorly. Additionally, maxillary sinusitis is observed. (c) Findings suggestive of acute inflammation due to odontogenic maxillary sinusitis (the arrow indicates the location). (d) MRI T2-weighted image showing inflammatory findings in the maxillary sinus, with a slightly high signal in the intraorbital fat.

Other ophthalmological tests, including visual acuity tests, fundus examinations, and intraocular pressure tests, were also performed, and all results were normal. CT and MRI imaging showed extensive orbital floor fracture with orbital herniation into the maxillary sinus and associated odontogenic maxillary sinusitis (Figure 2). The inferior rectus muscle was highly stretched and suspected to be partially attached to the orbital floor. Additionally, an MRI showed inflammation of the intraorbital fat.

**Figure 3:**
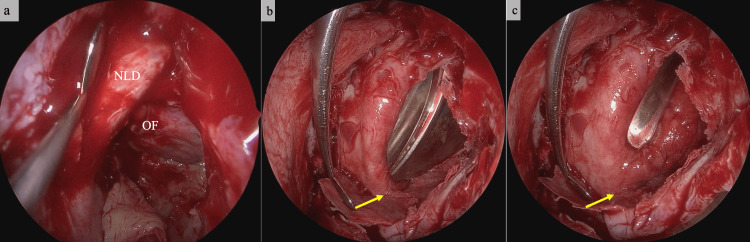
Intraoperative findings. The orbital floor was manipulated using a 70-degree endoscope through the lower eyelid. (a) The maxillary sinus mucosa is edematous and prone to bleeding. (b) The orbital floor was manipulated using a 70-degree endoscope through the lower eyelid. The orbital floor bone and surrounding tissue of the inferior rectus muscle were dissected from the lower eyelid. Adhesions are indicated by the yellow arrow. (c) The tissue surrounding the inferior rectus muscle after dissection. The scar tissue marked by the yellow arrow has been released. NLD: nasolacrimal duct; OF: orbital floor.

The suspected causes of both late-onset and acute-onset diplopia were tissue adhesion around the inferior rectus muscle, likely resulting from the spread of inflammation caused by acute maxillary sinusitis. The patient was examined by a neurologist, who ruled out diplopia caused by a central nervous system disorder. Subsequently, the patient gradually became aware of worsening diplopia. Prior to surgery, the patient underwent tooth extraction 140 days after the trauma. Surgery was performed 154 days after the trauma, which included endoscopic sinus surgery (ESS) and transconjunctival orbital floor repair. Intraoperatively, the maxillary sinus mucosa was severely edematous and prone to bleeding (Figure 3A). Adhesions between the tissue around the inferior rectus muscle and orbital floor were identified and sharply dissected (Figures 3B, 3C). Orbital reconstruction was achieved using a RapidSorb® implant (DePuy Synthes, Raynham, MA).

**Figure 4:**
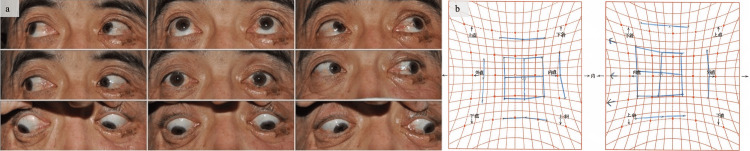
Postoperative findings of eye movement and enophthalmos. (a) Significant improvement is observed in the left enophthalmos and the upward movement restriction of the left eye. (b) Results of the Hess test after surgery: the upward gaze limitation of the left eye has significantly improved.

**Figure 5:**
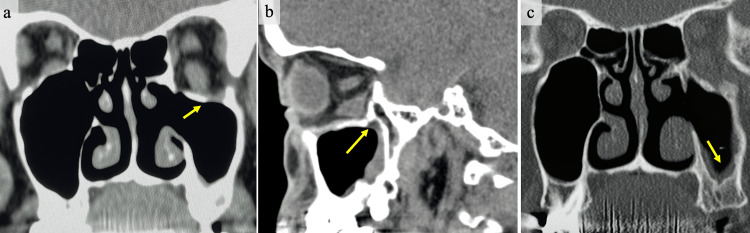
Postoperative CT findings. (a) A coronal CT scan shows that the orbital floor has been reconstructed, effectively containing the orbital contents within the orbit, as indicated by the yellow arrow. (b) A sagittal CT scan shows that the repair extends from the anterior to the posterior edge of the orbit, as indicated by the yellow arrow. (c) Odontogenic maxillary sinusitis improved following endoscopic sinus surgery and tooth extraction.

Eye movement, diplopia, and enophthalmos showed improvement early after surgery, and sufficient repair was confirmed by CT (Figures 4, 5A, 5B). The findings of sinusitis, including odontogenic maxillary sinusitis, also showed improvement (Figure 5C).

The patient's diplopia improved immediately postoperatively, with further resolution over 17 months. Residual mild superior diplopia persisted, but no recurrence of enophthalmos or diplopia was noted.

## Discussion

Diplopia in BOF improves gradually, while enophthalmos worsens gradually [6]. The causes of diplopia following BOF include soft tissue or muscle entrapment due to fracture, direct muscle trauma, hemorrhage within the orbit, muscle edema, and oculomotor nerve injury [7]. In addition, adhesion of the contents of the orbit can also cause diplopia [8]. Hinohira et al. reported that in cases of surgery for chronic BOF, adhesion to the periorbital tissue, bone fragments, and sinus mucosa were the cause of diplopia [9]. In most cases, diplopia appears shortly after trauma, making delayed onset rare. To our knowledge, this is the first reported case of diplopia developing suddenly after a two-month symptom-free interval. In this case, the primary cause of diplopia was the adhesion of the tissue around the inferior rectus muscle to the fractured orbital floor.

Inflamed and fibrotic tissue required sharp dissection during surgery (Figure 3). We believe that the adhesion of the tissues around the inferior rectus muscle was caused by the inflammation from odontogenic maxillary sinusitis spreading into the orbit. Ben Simon et al. reported that orbital cellulitis is a rare complication in patients with orbital floor fractures who have sinusitis. They speculate that bacteria from the paranasal sinuses adjacent to the orbit invade the orbit through the fracture site [10]. We believe that in this case, inflammation developed in the tissues around the inferior rectus muscle via the same pathway. Fortunately, it was identified when the patient began experiencing symptoms of diplopia, so we believe it did not progress to orbital cellulitis or an intraorbital abscess. Delayed diplopia in patients with orbital floor fractures may indicate intraorbital spread of sinusitis. Large orbital floor fractures do not heal spontaneously, and inflammation can spread from the adjacent paranasal sinuses, resulting in adhesion and scarring of the orbital tissue. This process can lead to sudden restriction of eye movement, as observed in this patient. In cases of extensive BOF accompanied by maxillary sinusitis with severe inflammation, such as odontogenic maxillary sinusitis, we believe that several months of follow-up observation is necessary to prevent late complications.

ESS was required not only for the repair of the orbital floor fracture but also for the treatment of odontogenic maxillary sinusitis. In cases of BOF with extensive bone fractures, enophthalmos can become a concern if rigid reconstruction is not performed [11]. Therefore, we performed maxillary sinus drainage, dissected the adhesions between the tissues around the inferior rectus muscle and the fractured orbital floor, and carried out rigid reconstruction using RapidSorb®.

There are many reports indicating that surgery for diplopia caused by BOFs is most effective in the early stages after the trauma. However, it has also been reported that diplopia improves in more than half of the cases, even after 30 days or more have passed since the trauma [12]. The surgery in this case was performed 154 days after the trauma, but it resulted in significant improvement in diplopia. Even if diplopia is caused by chronic BOF, surgical treatment should be considered.

## Conclusions

This case of BOF is extremely rare, as diplopia developed both late and suddenly. Although 154 days had passed since the trauma, the patient showed significant improvement following surgical treatment. The cause of the late and acute-onset diplopia is believed to be the spread of inflammation from odontogenic maxillary sinusitis into the orbit, resulting in adhesions in the tissues surrounding the inferior rectus muscle. Delayed onset of diplopia in BOF may serve as an early symptom of intraorbital inflammation. In cases of extensive BOF accompanied by paranasal sinusitis with severe inflammation, we believe that several months of follow-up observation is necessary to prevent late complications.
